# Classic biphasic pulmonary blastoma: A case report and review of the literature from 2000 to 2022

**DOI:** 10.1111/crj.13701

**Published:** 2023-09-29

**Authors:** Hui Yao, Xin Jiang, Ying Zeng, Xue Wang, Xuefeng Tang

**Affiliations:** ^1^ Department of Pathology Chongqing General Hospital Chongqing China

**Keywords:** chemotherapy, classic biphasic pulmonary blastoma, lung tumor, review, treatment

## Abstract

Classic biphasic pulmonary blastoma (CBPB), a distinct type of lung cancer, is a dual‐phasic tumor characterized by the co‐existence of low‐grade fetal adenocarcinoma and primitive mesenchymal stroma. Accounting for less than 0.1% of surgically removed lung cancers, CBPB commonly presents in individuals during their fourth to fifth decades of life, with smoking as a significant risk factor. The optimal management strategy entails surgical resection, supplemented by chemotherapy to improve prognosis. The frontline chemotherapeutic agents typically include platinum agents and etoposide, with preoperative neoadjuvant chemotherapy potentially enabling operability for initially inoperable cases. In recent years, targeted therapies, such as antiangiogenic agents, have emerged as promising new treatment strategies for CBPB. For patients exhibiting brain metastases or deemed inoperable, radiation therapy proves to be a crucial therapeutic component. CBPB prognosis is adversely affected by factors such as early metastasis, tumor size exceeding 5 cm, and tumor recurrence. In this regard, serological markers have been identified as valuable prognostic indicators. To exemplify, we recount the case of a 44‐year‐old female patient with CBPB, wherein serum lactate dehydrogenase levels showed significant diagnostic value. This report further incorporates a comprehensive review of CBPB literature from the past 22 years.

## Graphical abstract

Classic biphasic pulmonary blastoma (CBPB) represents an exceedingly rare and aggressively malignant subtype of sarcomatoid carcinoma. We recount the case of a 44‐year‐old female patient with CBPB, wherein serum LDH levels showed significant diagnostic value, and further incorporates a comprehensive review of CBPB literature from the past 22 years.

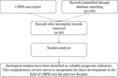

## 1 INTRODUCTION

Classic biphasic pulmonary blastoma (PB) (CBPB) represents an exceedingly rare and aggressively malignant subtype of sarcomatoid carcinoma. As outlined in the 5th edition of the World Health Organization's classification system, CBPB makes up less than 0.1% of all surgically resected lung cancers.^1^ Originally delineated by Barnett et al., CBPB exhibits distinctive biphasic histological characteristics, combining aspects of low‐grade fetal adenocarcinoma and primitive mesenchymal components. Unfortunately, this malignancy is notorious for its aggressive behavior and its associated poor patient survival rates.^2^ Currently, early surgical intervention remains to be the primary treatment strategy; however, a review of case reports over recent years suggests that improved prognosis has been achieved with new treatment modalities. This literature review aims to synthesize 22 years of developments, advancements, and alternative treatment methodologies relating to CBPB.

## 2 CASE REPORT

**FIGURE 1:**
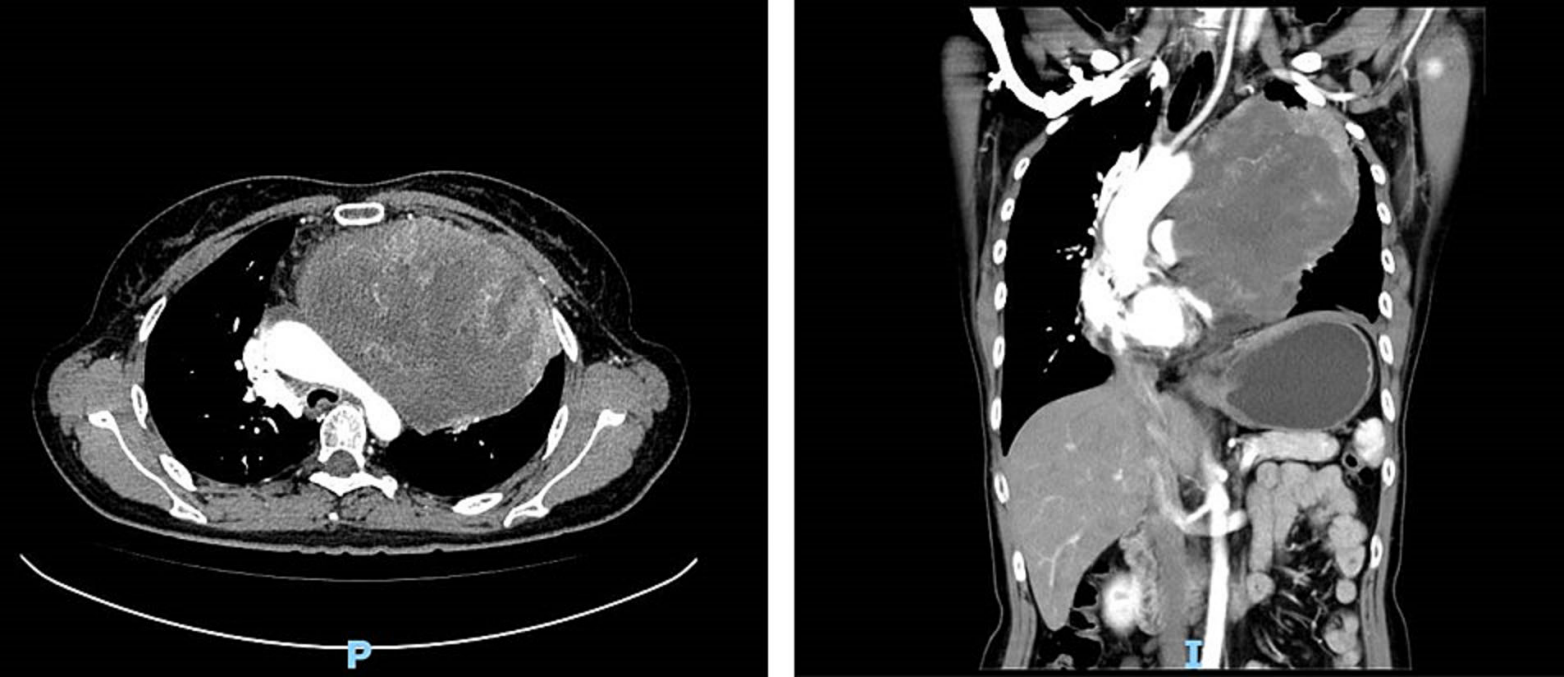
A contrast‐enhanced chest computed tomography scan revealing a large, heterogeneously enhancing, well‐circumscribed, oval mass located adjacent to the left middle and upper mediastinum. The mass, with dimensions of 15 × 12 × 14 cm, comprises both solid and cystic components.

A 44‐year‐old female, 170 cm tall and weighing 72 kg, with a 20‐year smoking history, averaging about 10 cigarettes per day, presented with a 2‐month history of cough and shortness of breath with exertion. No comorbidities were reported. Physical examination revealed dullness to percussion on the left side of the chest and reduced breath sounds during auscultation. Contrast‐enhanced computed tomography (CT) of the chest revealed a massive pericardial effusion and a large heterogeneously enhancing, well‐circumscribed, ovoid mass located adjacent to the left middle and upper mediastinum. The mass measured 15 × 12 × 14 cm, comprising both solid and cystic components (Figure 1). Neither mediastinal nor hilar lymph nodes were enlarged; however, the presence of bilateral pulmonary metastases was noted. Abdominal CT imaging revealed multiple hepatic lesions with a maximum diameter of 2 cm. Laboratory findings were notable for a markedly elevated serum lactate dehydrogenase (LDH) at 2009.4 IU/L (normal range 80–240 IU/L). Additional tumor markers have been tested, including alpha‐fetoprotein (AFP), carcinoma embryonic antigen, carbohydrate antigen (CA)‐153, CA199, CA125, CA724, serum ferritin, squamous cell carcinoma antigen, and neuron‐specific enolase all of which were within normal limits. A CT‐guided percutaneous biopsy subsequently revealed sarcomatoid carcinoma.

**FIGURE 2:**
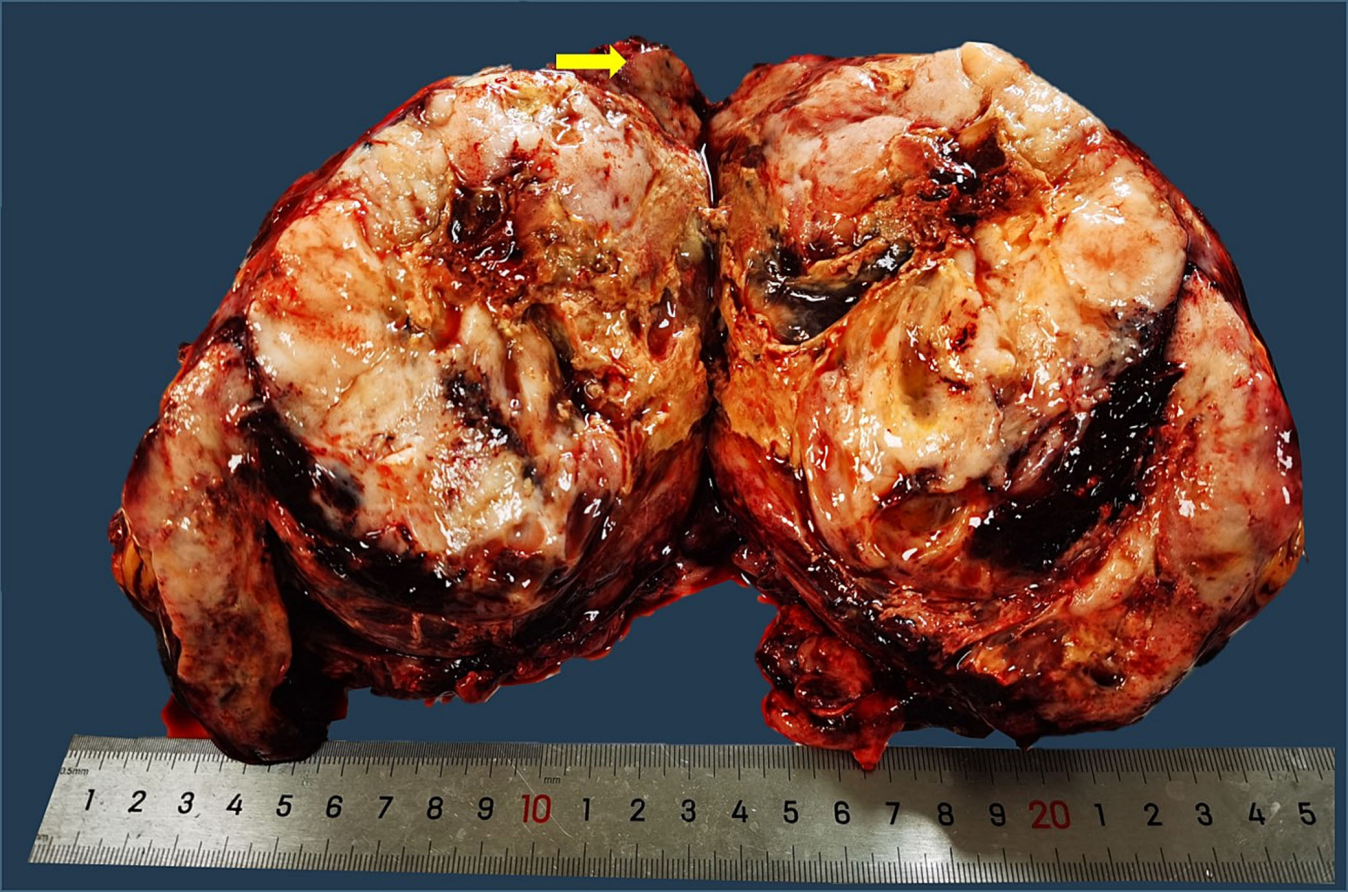
Gross examination of the surgically resected specimen, showing a well‐circumscribed, tan‐white, partially necrotic and hemorrhagic solid mass. Arrows indicate the surrounding residual lung tissue.

**FIGURE 3:**
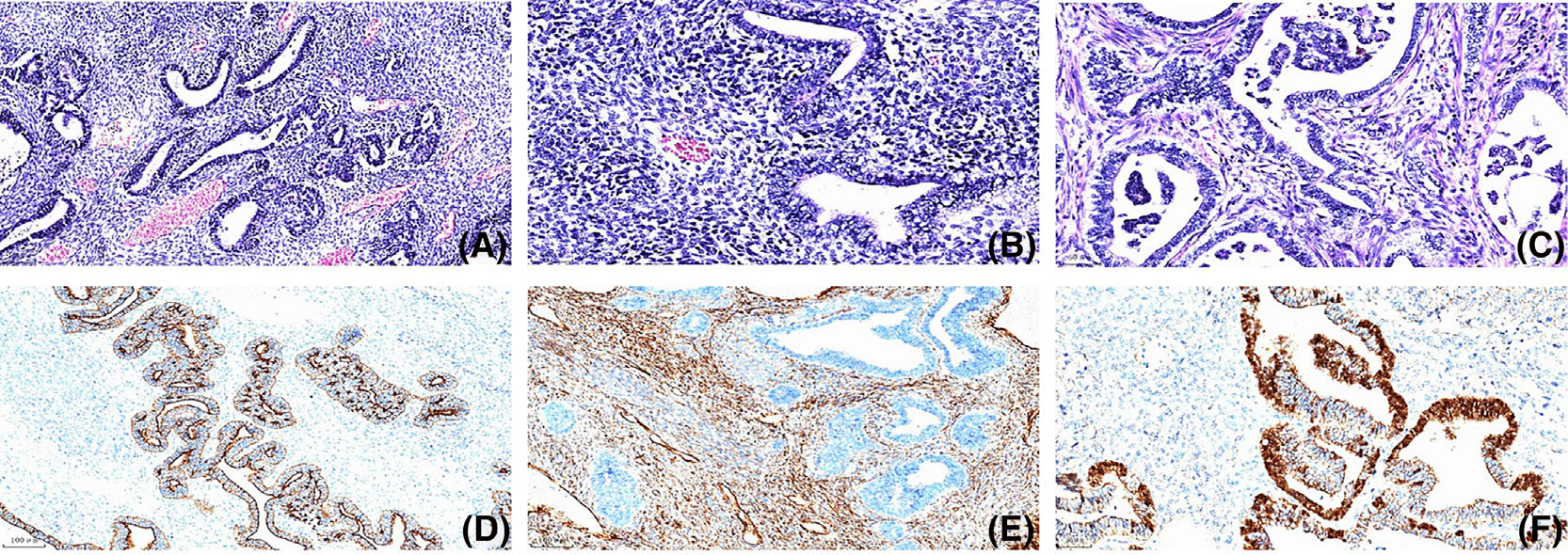
Histopathological and immunohistochemical analysis identifying mixed epithelial and mesenchymal malignancies. (A) Hematoxylin and eosin (H&E) staining at 10× magnification; (B, C) H&E staining at 20× magnification; (D) epithelial component expression of AE1/AE3; (E) identification of vimentin expression in the primitive mesenchymal stroma; (F) illustration of the predominantly nuclear/cytoplasmic localization of β‐catenin.

Given evident symptoms of compression, the patient underwent a series of surgical interventions including the resection of a mediastinal tumor, an anterior segmentectomy of the left upper lung, a wedge resection of the left lung, and a pericardiectomy. Inspection of the resected specimen revealed a 19‐cm, well‐circumscribed, tan‐white, solid mass with partial necrosis and hemorrhaging. Adjoining lung tissue was evident on the exterior of the mass (Figure 2). Histopathologic examination revealed a composite of low‐grade fetal adenocarcinoma intermixed with primitive mesenchymal stroma. Immunohistochemical examination showed epithelial cells that were positive for AE1/AE3, cytokeratin‐7, and thyroid transcription factor‐1 and that demonstrated both cytoplasmic and nuclear localization of β‐catenin. Moreover, the primitive mesenchymal component tested positive for vimentin (Figure 3). Final pathology report confirmed CBPB. Lymph nodes contained metastasis originating from a single‐phase epithelial component. Satellite nodules were discovered within the same lung, leading to a clinical staging of pT4N1M1 in accordance with the eighth‐edition TNM classification.

**FIGURE 4:**
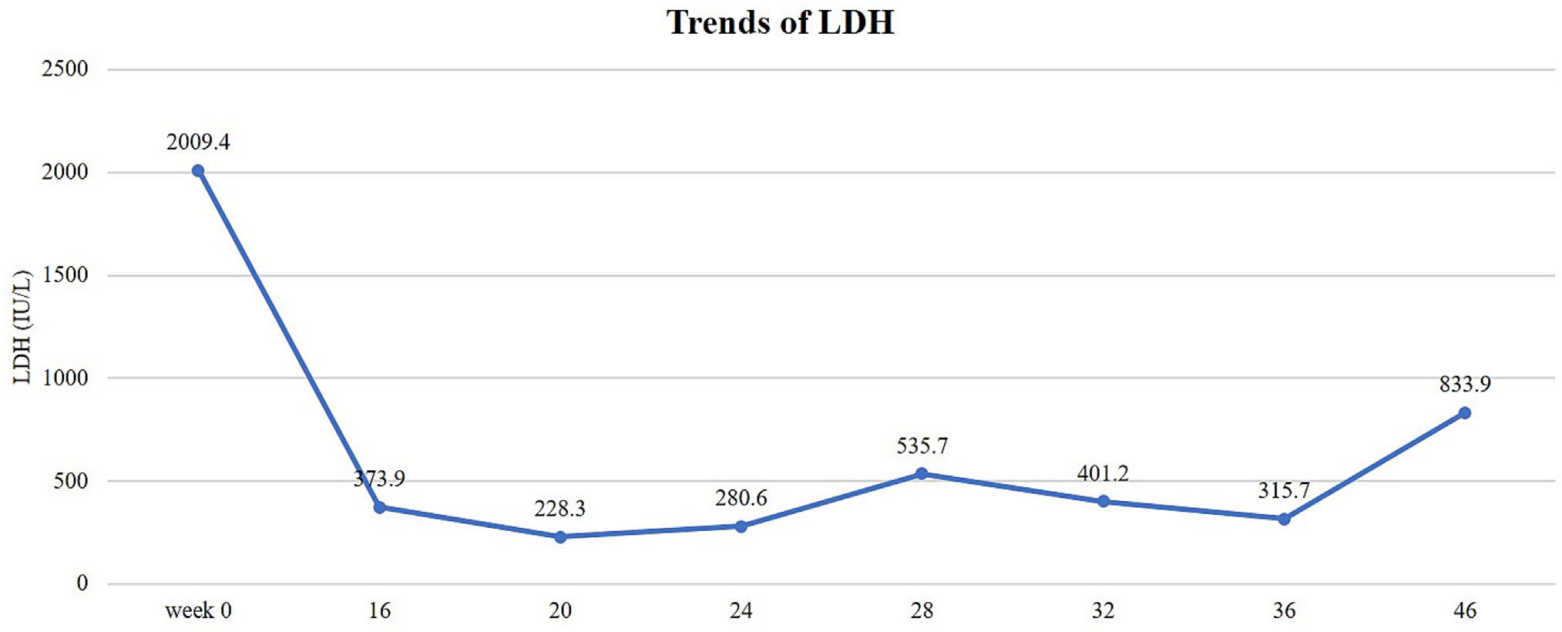
Clinical course depicting the fluctuating levels of lactate dehydrogenase (LDH) through various stages of treatment. Post‐lobectomy and adjuvant chemotherapy, LDH levels decreased. A spike in LDH levels was observed at 28‐weeks indicating recurrence. After one cycle of a combined chemotherapy regimen (dacarbazine, vindesine, cyclophosphamide, epirubicin, and anlotinib), LDH concentrations declined once more. However, upon deterioration of the patient's condition after 10 weeks, the LDH level increased again.

Subsequent treatment consisted of a single cycle of adjuvant chemotherapy, incorporating pemetrexed disodium at a dose of 600 mg/m^2^, lobaplatin at 40 mg/m^2^, and bevacizumab at 600 mg/kg. After one cycle of chemotherapy, a CT scan revealed an increase in soft tissue shadows within the operated region, suggestive of a recurrence. The patient subsequently received a regimen of epirubicin at 70 mg/m^2^ on Day 1, ifosfamide at 1 g/m^2^ (Days 1–3), and oral anlotinib at 12 mg (Days 1–14). After receiving chemotherapy with epirubicin and ifosfamide, the patient experienced mild nausea, vomiting, poor appetite, and abdominal bloating. A follow‐up evaluation after 4 weeks indicated significant reduction in the recurrent lesions within the operation site. After three cycles of this chemotherapy regimen, the serum LDH level notably decreased from 2009.4 to 228.3 IU/L (Figure 4).

At the 2‐month follow‐up, CT imaging revealed new lesions in the pelvic and thoracic spine suggestive of metastatic disease. The serum LDH level had also risen to 535.7 IU/L (Figure 4). The patient then received three additional cycles of adjuvant chemotherapy comprising of vindesine at 2 mg/m^2^, cyclophosphamide at 1 g/m^2^, and epirubicin at 70 mg/m^2^ on Day 1; dacarbazine at 0.4 g/m^2^ (Days 1–3); and oral anlotinib at 12 mg (Days 1–14). This regimen led to a reduction in the serum LDH level to 315.7 IU/L (Figure 4). However, after 3 months, the patient's clinical condition deteriorated, and the serum LDH escalated to 833.9 IU/L (Figure 4). The patient died of respiratory failure several days later.

## 3 DISCUSSION

PB, recognized as a rare variant of sarcomatoid carcinoma, is characterized by its rapid progression and adverse prognosis. The scarcity of effective treatment strategies is a significant challenge, which is compounded by the inability to conduct extensive, multicenter clinical trials due to its rarity. Consequently, the current management of PB is predominantly based on isolated case studies and accumulative knowledge garnered over decades across multiple centers.

Diagnosing CBPB typically does not pose significant difficulties. In the case under consideration, a histopathological examination revealed a mix of low‐grade fetal adenocarcinoma and primitive mesenchymal stroma. Further, immunohistochemical investigation confirmed the cytoplasmic and nuclear presence of β‐catenin, thereby corroborating the CBPB diagnosis.

The differential diagnosis includes fetal adenocarcinoma and carcinosarcoma. Fetal type adenocarcinoma is a monophasic tumor without primitive mesenchymal components. Carcinosarcoma with an adenocarcinoma component resembling high‐grade fetal adenocarcinoma is often at risk of being misdiagnosed as CBPB. In distinguishing these entities, the immunohistochemistry findings (notably the predominantly membranous expression of β‐catenin and the absence or reduction of TTF1 expression) may prove to be instrumental.^3^

PB was first reported by Barrett and Barnard in 1945.^4^ The subsequent work by Larsen and Sorensen included a review of the clinicopathological characteristics of 202 cases from 1962 to 1995, encompassing 156 instances of PB.^5^ Van Loo et al. conducted an extensive review of literature from 1995 to 2011, identifying 42 cases of CBPB. Their work offers a comprehensive summary of the clinicopathological attributes, diagnosis, and treatment modalities for CBPB.^4^ Additional studies have provided valuable insights into PB's treatment approaches, metastasis patterns, and prognostic indicators.^3^, ^6^, ^7^, ^8^, ^9^

In this comprehensive review, we collated data from 66 reported cases of CBPB spanning a period of 22 years, from 2000 to 2022, focusing on recent advancements pertaining to clinical characteristics, genetic mutations, treatment modalities, and prognosis (Table 1). Remarkably, several instances showcased unexpectedly favorable outcomes. As established in previous studies, radiotherapy offers significant benefits for patients presenting with brain metastases. With increasing frequency, serological markers are being utilized to predict prognosis and monitor disease progression. For instance, our data demonstrated that serum LDH levels correlate with disease severity. However, it is worth noting that we excluded certain reports in non‐English languages and cases with undisclosed survival durations. This comprehensive review serves to encapsulate the latest developments in the field of CBPB over the past two decades.

**TABLE 1:** Overview of cases of pulmonary blastoma between 2000 and 2022.

Pat no.	Type	Age (years)	Sex	Smoking	Size (cm)	Lobe	Tumor biomark	Stage	Metastasis	Genetic mutation	Surgery	CT	RT	Outcome (months)	Refs.
1	CBPB	30	M	Y	10.8	RPB		T4N2M0	Paratracheal lymph node		NA	PDD, VP‐16		Alive wr 2	^2^
2	CBPB	58	M	Y	5	RUL	AFP	T3N0M0	Liver	EGFR	Lob + lym	Ned, Pac		Alive wr 48	^6^
3	CBPB	60	F	?	?	LUL			Brain		Lob			Alive wr 6	^8^
4	CBPB	64	F	Y	3	RUL		T1N0M0			Lob	MM‐C		DOD 8	^10^
5	CBPB	22	F	N	10	RLL		T4N0M0				Vcr, Act, Cyclo		Alive wr 6	^11^
6	CBPB	50	F	Y	13.5	RUL		T4N0M0			Lob + seg	PDD, VP‐16		Alive wr 4	^12^
7	CBPB	7	F	?	12	R??		T4N0M0		β‐catenin	Pne	PDD, VP‐16		Alive wr 12	^13^
8	CBPB	3	M	?	8	R??		T4N0M0			Pne			Alive wr 6	^14^
9	CBPB	36	F	Y	10	LLL		T4N0M0		ROS1	Lob	PDD, Cri		DOD 7	^15^
10	CBPB	33	F	Y	7	LLL		T3N0M0			Lob + lym			Alive wr 12	^16^
11	CBPB	44	F	Y	5.8	LUL		T3N1M1		ROS1		Cri		Alive recurrence 6	^17^
12	CBPB	71	F	Y	7	RLL		T3N0M1	Vertebra		Lob	PDD, VP‐16, Carbo	30 Gy	Alive wr 84	^18^
13	CBPB	66	F	Y	8	LLL		T4N0M1			Lob + lym	PDD, VP‐16,		DOD 6	^19^
14	CBPB	38	F	Y	9.5	LUL		T4N2M1	Brain		Lob + lym	PDD, Vin, Doc	50.4 Gy	Alive wr 120	^7^
15	CBPB	29	F	N	9	LLL		T4N1M1	Ovary		Lob + lym	PDD, If, VP‐16	59.49 Gy	Alive wr 120	^7^
16	CBPB	29	F	?	?	RML					Lob + lym	Ned	Exist	Alive wr 6	^20^
17	CBPB	68	M	Y	?	LUL					NA		Exist	Alive wr 1	^21^
18	CBPB	62	M	?	5.3	LUL		T3N1M0			Lob	PDD, If	64 Gy	Alive wr 26	^22^
19	CBPB	67	M	Y	9	RLL		T4N0M0			Pne			Alive wr 23	^23^
20	CBPB	75	M	Y	2	R??		T1N1M1	Brain		Frontal lobectomy			dead unrelated 5 days	^24^
21	PB	37	F	N	?	LLL		T3N2M0			Lob + lym	Exist	Exist	Alive wr 36	^25^
22	CBPB	58	M	?	?	?					Pne			Alive recurrence 24	^26^
23	CBPB	19	F	N	4	LUL		T3N2M1	Brain		Occipital lobectomy			DOD 1.5	^27^
24	CBPB	68	M	?	?	LUL	AFP, NSE, CEA	T3N0M0	Liver, spleen			Pac, Bev, Pem, Doc		DOD 10	^28^
25	CBPB	68	M	Y	10.5	LUL		T4N0M0	Brain, liver		Lob + lym	Exist	Exist	DOD 6	^29^
26	CBPB	29	F	?	?	?				DICER1		PDD, ADM, Cyclo		Alive wr 4	^30^
27	CBPB	25	F	Y	5.5	LUL		T4N0M0		PD‐L1	Pne	PDD, VP‐16	50.4 Gy	Alive wr 8	^31^
28	CBPB	73	F	?	5	RUL		T2N0M0			Lob			Alive wr 48	^32^
29	CBPB	17	M	?	12	RUL, RML		T4N?M1	Bone		Lob	PDD, VP‐16,	Exist	Alive wr 24	^33^
30	CBPB	43	M	N	6.7	?	AFP, β‐HCG	T3N0M0			NA	PDD, If, VP‐16,	40 Gy	Alive wr 3	^34^
31	CBPB	22	F	?	12	?		T4N0M1	Liver, bone		Pne	If, ADM Carbo, Vcr, Act, Gem	40 Gy	DOD 26	^35^
32	PB	62	M	Y	10.7	LUL		T4N2M1	Adrenal gland, scrotum		Lob	Carbo, ADM, Cyclo		DOD 2	^36^
33	CBPB	70	M	?	7	RUL		T3N0M0			Lob + lym			Alive wr 36	^37^
34	CBPB	18	M	?	17	R??		T4N?M?			NA	PDD, ADM, Vcr, VP‐16		Alive wr 3	^38^
35	CBPB	16	F	?	11	RUL		T4N?M0				Carbo, If, VP‐16		Alive wr 3	^39^
36	CBPB	51	M	Y	12.9	LUL		T4N?M?			Pne			Alive wr 6	^40^
37	CBPB	77	M	Y	6	RUL		T3N0M0			Lob + lym	Sor		DOD 12	^4^
38	PB	45	F	N	12	LUL		T4N0M0			Lob	Exist		Alive wr 8	^41^
39	CBPB	36	M	Y	13	?	AFP, β‐HCG	T4N0M0			NA	BLM, PDD, VP‐16, Pac, If	25 Gy	DOD 15	^42^
40	CBPB	75	M	?	8	RUL	CEA	T3N0M0			Lob + lym		Exist	DOD 7	^43^
41	PB	24	M	?	7.7	RUL		T4N0M0			Lob	PDD, VP‐16	50 Gy	Alive recurrence 60	^44^
42	PB	New‐born	M	N	7	RUL		T1N0M0			Lob			Dead unrelated several days	^45^
43	CBPB	38	F	?	?			T?N?M1	Ovary		Lob	PDD, VP‐16		Alive recurrence 6	^46^
44	CBPB	47	F	N	15	LUL		T4N0M0			Pne	Carbo, VP‐16, If,	6000 Gy	Alive wr 36	^47^
45	CBPB	40	F	Y	4.6	RUL		T2N2M0			Lob	Exist	Exist	Alive wr 14	^48^
46	CBPB	58	M	?	10	RUL	AFP	T4N0M0			Lob + lym	PDD, VP‐16	50 Gy	Alive wr 70	^9^
47	CBPB	73	F	?	3.5	LLL		T2N0M0			Lob + lym			Alive wr 30	^49^
48	CBPB	73	M	?	7	RLL		T3N0M0			Lob + lym	Exist		Alive wr 36	^50^
49	CBPB	52	F	?	?	RUL				β‐catenin, P53	Lob			Alive recurrence 7	^51^
50	CBPB	71	M	Y	6.5	LLL		T3N?M1				PDD, I‐HCI	Exist	Alive recurrence 2	^52^
51	CBPB	78	M	Y	6.5	RLL	LDH	T3N0M0			Lob			Alive wr 12	^53^
52	PB	84	M	Y	11.5	LLL		T4N0M0			Lob + lym			DOD 6	^54^
53	CBPB	37	F	?	8	RUL		T4N?M1	Kidney		Lob + lym	Carbo, Gem, Pem, Bev, Sor	4800 cGy	Alive wr 36	^55^
54	CBPB	79	M	Y	?	RUL		T2N1M0			Lob			Alive wr 12	^56^
55	PB	41	F	Y	?	?		T?N?M1			Lob		Exist	DOD 4	^57^
56	CBPB	51	M	?	10	?		T4N?M1			Lob		45 Gy; 30 Gy; 15 Gy	DOD 24	^58^
57	CBPB	63	M	Y	15.8	?		T4N0M0			Lob	Cyclo, Doxo, Vcr		Alive wr 4	^59^
58	CBPB	16	M	?	?	?					NA	PDD, VP‐16		DOD 12	^60^
59	PB	43	F	?	?	LLL						PDD, Gem, I‐HCI, VP‐16	20 Gy	DOD 72	^61^
60	CBPB	56	M	N	19	RUL	AFP	T4N0M0			Lob	PDD, VP‐16, If, Vin,		DOD 11	^62^
61	CBPB	48	M	Y	16	RUL		T4N0M0			Lob	Epi, If, Gem, Carbo, Vin,		DOD41	^63^
62	PB	49	M	Y	?	?	LDH				NA	Cyclo, Doxo, VP‐16, PDD	20 Gy	Alive wr 12	^64^
63	CBPB	53	M	Y	17	LLL		T4N0M0			Lob	VP‐16, PDD		DOD 12	^65^
64	CBPB	72	M	Y	8.5	RLL		T4N0M0			Lob		50 Gy	DOD 12	^65^
65	PB	12	M	?	15	LLL		T4N0M0			Lob	Exist	Exist	Alive recurrence 18	^66^
66	CBPB	3	M	?	12	RUL		T4N0M0				Vcr, VP‐16, If, Me, ADM		DOD12	^67^

Abbreviations: Act, actinomycin‐D; ADM, doxorubicin; Alive wr, alive without recurrence; Bev, bevacizumab; BLM, bleomycin; Carbo, carboplatin; CEA, carcinoma embryonic antigen; Cri, crizotinib; CT, chemotherapy; Cyclo, cyclophosphamide; Doc, docetaxel; DOD, death of disease; Doxo, doxorubicin; Epi, epirubicine; Gem, gemcitabine; If, ifosfamide; I‐HCI, irinotecan hydrochloride; LLL, left lower lobe; lob, lobectomy; LUL, left upper lobe; lym, lymphadenectomy; Me, mesna; MM‐C, mitomycin‐C; Ned, nedaplatin; Pac, paclitaxel; PDD, cisplatin; Pem, pemetrexed; per, pericardiectomy; pne, pneumonectomy; RLL, right lower lobe; RML, right middle lobe; RPB, right principal bronchus; RT, radiotherapy; RUL, right upper lobe; seg, segmentectomy; Sor, sorafenib; Vcr, vincristine; Vin, vinorelbine; VP‐16, etoposide.

The clinical manifestations of CBPB bear a striking resemblance to those observed in other non‐small cell lung carcinomas. Our review substantiates previous findings, with the most frequently reported symptoms being cough (33%), chest pain (33%), and hemoptysis (29%). A notable case report by Tehrani et al. described frequent hypoglycemic episodes in a CBPB patient, which resolved post‐surgery. This may be attributed to tumor cell secretion of insulin‐like growth factors.^26^

Existing literature, such as the report by Koss et al., suggests a higher prevalence of CBPB in individuals during their fourth to fifth decades of life, with no apparent gender disparity.^68^ Our analysis aligns with these findings, reporting a median age of 49 years and a marginally higher incidence in males (37) as compared with females (29). A study by Van Loo et al., involving 42 CBPB cases, found an average tumor size of 9.1 cm in the largest diameter. Our data present a comparable result, with an average tumor size of 9.4 cm. Moreover, our findings highlight a propensity for CBPB to occur predominantly in the upper lobes of the lung. A majority of cases in our series presented with lesions in the upper lobe of the lung (right upper lobe, 47.5%; left upper lobe, 32.5%), a trend mirroring the observations of Van Loo et al.

The relatively low incidence of CBPB often results in an obscured understanding of its pathogenesis. However, smoking has been solidly established as a risk factor,^68^ as supported by our case study in which the patient was a chronic smoker. Further confirmation is provided by our review, showing that a notable 77% (30 out of 39) of CBPB patients had a history of smoking. This significant percentage substantiates the correlation between smoking and CBPB. Previous studies indicate that CBPB frequently exhibits missense mutations in exon 3 of the *CTNNB1* gene. These mutations subsequently lead to an abnormal nuclear localization of the β‐catenin protein, thereby activating the WNT pathway.^69^ Tian et al. investigated the effects of cigarette smoke extract on β‐catenin through in vitro experiments. The results demonstrated a positive correlation between the expression of β‐catenin in the nucleus and cytoplasm and the concentration of cigarette smoke extract. Therefore, abnormal expression of β‐catenin may be a potential mechanism through which smoking leads to CBPB.^70^, ^71^ Other mutations linked to CBPB involve *TP53*, *ROS1*, and *EGFR*.^6^, ^15^, ^72^ In a report by de Kock et al., two cases of adult‐onset CBPB with somatic mutations in *DICER1* and *CTNNB1* were presented. This led them to postulate a probable association between adult‐onset CBPB and somatic mutations of *DICER1*.^30^

Other gene alterations specific to CBPB have also been reported. Kim et al. presented a case of CBPB in a patient with a history of Stage IV lung adenocarcinoma. They noted that this case was characterized by a high tumor burden and exhibited a distinctively different gene mutation profile compared with the primary lung adenocarcinoma. Specifically, they found extensive mutations in the *NF1*, *FGFR3*, *MBN1*, *ATRX*, *KDMGA*, *PARK2*, and *PBRM1* genes in the CBPB, which were not observed in the primary adenocarcinoma.^73^ In a separate study, Zhao et al. compiled a retrospective analysis of 16 PB patients, with an average age of 40 years. The analysis revealed that mutations were identified in 9 of the 56 genes profiled, namely, *BRCA2*, *ERBB4*, *ALK*, *MET*, *BRAF*, *RAF1*, *PTEN*, *EGFR*, and *PIK3CA*.^74^ Moreover, Bosch‐Barrera et al. reported a unique case with high expression of PD‐L1 (>90% of tumor cells). This suggests a potential utility of immunotherapy in treating similar cases in the future.^31^

The precise histogenesis of CBPB continues to be a subject of inquiry. A case presented by Hansen et al. underscored the significance of the stem cell factor receptor KIT's expression in both the epithelial and mesenchymal components of CBPB. This finding implies that the CBPB may originate from a pluripotent cell capable of differentiating into both stromal and epithelial morphologies.^75^ In another noteworthy study, Takahashi et al. conducted a comprehensive examination of the genetic alterations accumulated in the epithelial and mesenchymal components of the primary tumor and brain metastases of CBPB.^51^ Their findings revealed a commonality of allelic imbalance in chromosome regions 17p11‐p13 and 14q24‐q32, along with a β‐catenin mutation, across all three components. Nonetheless, they also identified unique genetic alterations that differentiated the epithelial and mesenchymal components. Specifically, the allelic imbalance of chromosomes 6p24‐p25 and 6q14‐q27 was exclusive to the epithelial component, while the mesenchymal component featured an allelic imbalance of chromosomes 3p11‐p14 and 9p21‐p24, as well as a mutation of the *p53* gene. The culmination of these results supports the hypothesis of a single cell origin for CBPB. Furthermore, they underscore the likelihood that the biphasic nature of this tumor is predominantly a result of disparate accumulations of genetic alterations in the epithelial and mesenchymal cells.^51^

CBPB typically exhibits rapid progression, with a majority of patients presenting at an advanced stage. This observation is validated by a study conducted by Kim et al., in which the tumor volume of a CBPB patient was reported to have amplified eightfold within a span of merely 2 months.^73^ Hence, timely surgical intervention is recommended as the primary treatment for CBPB.^7^ In a comparative analysis conducted by Francis and Jacobsen, a significant survival difference was observed between patients who underwent surgery and those who did not. The mean survival time reported was 33 months for the 66 resected cases, in contrast to a mere 2 months for the 17 unresected cases.^76^ A separate review by Zaidi et al. of six CBPB patients revealed a similar trend. Out of the six, four patients who underwent resection are still alive, with a median survival time of 43.5 months. However, the remaining two patients who did not undergo resection died within a median of 5.5 months.^77^ One patient who received a wedge resection of the lobe, without subsequent chemotherapy or radiation, survived for 4 years.^32^

The identification of specific serum markers for CBPB, however, remains elusive.^6^ Previous studies have noted fluctuations in the serum levels of AFP (reported in 7 cases), β‐human chorionic gonadotropin (β‐HCG, reported in 3 cases), and LDH (reported in 3 cases), corresponding to the improvement or recurrence of the disease.^6^, ^9^, ^26^, ^28^, ^42^, ^53^, ^62^, ^64^ In line with these findings, our research demonstrated an abnormal elevation in LDH levels, which, following surgical and chemotherapy interventions, rapidly decreased. Thereafter, the LDH levels were found to either decrease or increase, paralleling the respective improvement or recurrence of the disease.

LDH plays a pivotal role in the glycolytic pathway, a metabolic process integral to malignancies. Prior research has indicated a significant association between elevated serum LDH levels and adverse prognosis in various malignancies.^78^ Such elevation is frequently ascribed to increased tumor burden and altered cancer metabolism.^79^ In their retrospective study, Wang et al. analyzed a cohort of 224 patients diagnosed with lung cancer that had metastasized to the brain. Their findings suggested that elevated serum LDH could be predictive of a poorer prognosis in patients with NSCLC with brain metastases.^78^ A different investigation led by Hou et al. suggested that LDH could stimulate tumor progression by modulating epithelial–mesenchymal transition‐related molecules.^80^ Additionally, Ding et al. confirmed the role of LDH in promoting resistance to chemotherapy, radiotherapy, and targeted therapy by enhancing immunosuppression within the tumor microenvironment.^81^ Given these findings, we posit that serum LDH concentrations may serve as a valuable prognostic biomarker for CBPB. Further, LDH measurements may prove to be a beneficial tool in monitoring both the response to chemotherapy and overall prognosis.

CBPB has a poor prognosis and requires multidisciplinary collaborative treatment to improve survival rates.^82^ At present, a standard chemotherapy regimen for CBPB remains undefined. The work of Francis and Jacobsen noted that the response rate to initial chemotherapy in assessable patients stands at 26%.^76^ It has been suggested that regimens incorporating a platinum agent and etoposide (VP‐16) could yield comparatively favorable responses. In a comprehensive review of chemotherapy regimens documented in the literature, Oshika et al. noted that the majority of CBPB patients with survival exceeding 1 year were treated with chemotherapy regimens comprising a platinum agent, VP‐16, or both.^9^ Table 1 presents an exhaustive list of chemotherapeutic agents employed in the treatment of PB patients, as evidenced in studies spanning the past two decades. Platinum agents (32/39) and VP‐16 (23/39) were found to be most prevalent among these. Moreover, among patients who survived for more than a year, the platinum agent (18/20) and VP‐16 (13/20), or a combination of both (12/20) were the treatments of choice. Consider the patient who underwent three cycles of cisplatin and VP‐16, followed by a cycle of carboplatin and VP‐16 and subsequent radiation therapy who was still alive 7 years post‐treatment.^18^ In another instance, a CBPB patient with brain metastases who received four cycles of cisplatin and vinorelbine followed by radiation therapy was reported to be alive a decade later.^7^ Equally notable was the case of a patient with bilateral ovarian metastases who successfully gave birth 1 year after undergoing four cycles of cisplatin, ifosfamide, and VP‐16, followed by radiation therapy, and was alive 10 years after the treatment.^7^ Aside from these, additional chemotherapeutic agents such as ifosfamide, doxorubicin, docetaxel, cyclophosphamide, vincristine, paclitaxel, and sorafenib have also been used in the treatment of CBPB.

Consideration should also be given to targeted therapies, including antiangiogenic agents, as a novel treatment strategy for CBPB. Mulamalla et al. noted a rare incidence of renal metastasis of biphasic PB that demonstrated a positive response to sorafenib, an oral multikinase inhibitor.^55^ Another case showcased the potential of an oral multikinase inhibitor, anlotinib, in treating CBPB.^6^

The role of adjuvant chemotherapy, however, remains unclear. Zagar et al. detailed an instance in which a patient diagnosed with CBPB underwent left upper lobectomy without chemotherapy or radiation, only to present with a right lung mass 5 years later. After administration of two cycles of cisplatin and VP‐16 along with radiation therapy, significant disease regression was observed on a chest CT 3 weeks later. The patient subsequently underwent pneumonectomy and recovered well.^44^ In another instance, Zaidi et al. report two distinct cases in which the disease was effectively downstaged following neoadjuvant chemotherapy.^77^

Radiotherapy has demonstrated efficacy in the management of metastases in patients with PB, particularly those with brain metastases. In a study collating all 13 reported instances of CBPB with brain metastases, Park et al. found that two patients who underwent local radiotherapy showed a positive response.^8^ In most centers, radiotherapy is employed for cases that are unresectable, locally advanced, or for those with metastatic disease seeking palliative care.^4^

The prognosis for PB remains bleak. Approximately two‐thirds of patients succumb to the disease within 2 years of diagnosis, 16% survive up to 5 years, and only 8% survive a decade.^68^ In our retrospective analysis, we discovered that the median survival time for the 66 CBPB patients under review was 1 year, with only 6 (or 9%) surviving past the 5‐year mark. Intriguingly, these findings diverge slightly from the statistics reported by Koss et al. The most commonly reported sites of CBPB metastasis are the brain and liver, although metastatic progression can also be observed in the bones, spine, adrenal glands, and ovaries. Of particular note is the tendency for PB metastases to present in a uniphasic, rather than biphasic, pattern, with the epithelial component being more frequently involved in metastasis. This was also observed in our case study, in which the lymph node metastases exhibited an epithelial component. The factors portending a poorer prognosis for CBPB include early metastasis, large tumor size (greater than 5 cm in diameter), and tumor recurrence.^4^

Despite a pressing need, mechanistic studies on CBPB remain scarce. However, the establishment of the first human PB cell line by Camerlingo et al. has helped to pave the way for future research in this area.^83^

## 4 CONCLUSION

CBPB is a rare subtype of sarcomatoid carcinoma, characterized by its rapid progression and poor prognosis. Patients commonly present with cough, chest pain, and hemoptysis. Current literature primarily consists of isolated case studies and reports involving small cohorts, with a notable absence of comprehensive, large‐scale investigations. Surgical resection and chemotherapy comprise the preferred treatment strategies for CBPB, with platinum and VP‐16 forming the first‐line chemotherapeutic agents of choice. The incorporation of targeted therapies, such as antiangiogenic agents, could potentially represent a novel avenue for CBPB management. Furthermore, preoperative neoadjuvant chemotherapy might render certain inoperable cases amenable to treatment. Regarding residual disease and metastases, radiotherapy exhibits enhanced effectiveness, particularly in cases with cerebral metastases.

In summary, our review encapsulates all reported cases of CBPB over the last 22 years, with the hope of contributing valuable insights toward the clinical diagnosis and management of this condition.

## AUTHOR CONTRIBUTIONS

Hui Yao: Substantial contributions to conception and design; collecting and assembling materials; analysis and interpretation of available data and drafting the manuscript; and finalizing the version to be published. Xuefeng Tang: Substantial contributions to conception and design; analysis and interpretation of available data; finalizing the version to be published. Xin Jiang and Ying Zeng: Analysis and interpretation of available data; revising the article critically for important intellectual content. Xue Wang: revising the article critically for important intellectual content. All authors reviewed and approved the final version of the manuscript.

## CONFLICT OF INTEREST STATEMENT

The authors declare that there are no conflicts of interest. No benefits in any form have been received or will be received from a commercial party related directly or indirectly to the subject of this article.

## ETHICS STATEMENT

This study protocol was reviewed and approved by the Ethics Committee of the Chongqing General Hospital.

This study was obtained written informed consent from participants for publication of the details of their medical case and any accompanying images.

## Data Availability

Data sharing is not applicable to this article as no new data were created or analyzed in this study.
